# Assessment of patient safety culture among healthcare employees in major Eswatini public hospitals

**DOI:** 10.1371/journal.pone.0325292

**Published:** 2025-06-25

**Authors:** Mpumelelo Goodwill Ndlela, Chia-Hui Wang, Nai-Wen Kuo, Bongani Sibandze, Qhubekani Mpala, Victor Williams

**Affiliations:** 1 School of Healthcare Administration, College of Management, Taipei Medical University, Taipei, Taiwan; 2 Access to Medicine, Clinton Health Access Initiative, Mbabane, Kingdom of Eswatini; 3 Doctoral Program in International Health, College of Medicine, National Yang-Ming Chiao-Tung University, Taipei, Taiwan; 4 Department of Real Estate & Built Environment, College of Public Affairs, National Taipei University, New Taipei City, Taiwan; 5 Research Center for Sleep Medicine, College of Medicine, Taipei Medical University, Taipei, Taiwan; 6 Bachelor’s Program in Smart Sustainable Development and Management, International College of Sustainability Innovations, National Taipei University, New Taipei City, Taiwan; 7 Faculty of Health Sciences (Manzini Campus), Southern Africa Nazarene University, Manzini, Kingdom of Eswatini; 8 Unit of Epidemiology and Biostatistics, School of Public Health, Faculty of Health Sciences, University of the Witwatersrand, Johannesburg, South Africa; University of Hafr Al-Batin, SAUDI ARABIA

## Abstract

This primary study assessed the patient safety culture in five major public government hospitals in Eswatini. A cross-sectional survey was conducted among hospital employees using the validated Hospital Survey on Patient Safety Culture (HSOPSC) developed by the Agency for Healthcare Research and Quality (AHRQ). The average positive patient safety culture (PSC) score was 43%. The highest-rated composite was “teamwork within units” (75%), while the lowest-rated was “staffing” (25%). All 11 PSC composites scored below the 2018 AHRQ survey benchmarks. These findings underscore the need for significant improvements in PSC across Eswatini’s hospitals. Additionally, the study revealed a lack of robust patient safety reporting systems in the five hospitals, which hinders efforts to address safety issues and reduce medical errors.

## Introduction

Medical errors are common in healthcare facilities [1], and the World Health Organization (WHO) reports that unsafe healthcare practices result in approximately 2.6 million deaths each year in low- and middle-income countries alone [2], with 5–15% of hospital admissions involving medical errors [3]. Tens of millions of patients worldwide suffer disabling injuries or death each year directly attributed to unsafe medical practices and care [4].

In the Eastern Mediterranean and African regions, nearly one-third of patients who experience an adverse event (AE) die; 14% sustain a permanent disability, 16% a moderate disability, 30% a minimal disability, and in 8% of cases, the extent of harm cannot be identified [5]. Medical errors raise serious concerns about patient safety among healthcare workers and can be reduced by cultivating a stronger and more supportive patient safety culture (PSC). PSC is a crucial component of healthcare quality that requires serious attention and improvement to ensure high-quality patient care [6]. Hospital staff—especially physicians and nurses involved in primary care—play a vital role in maintaining and promoting patient safety due to the nature of their responsibilities [7].

Patient safety is a critical aspect of healthcare quality in developing countries, often impacted by a range of developmental challenges [8,9]. Eswatini, as a developing nation, has seen elevated rates of reported medical errors, which undermine total quality management in healthcare facilities and contribute to patient fatalities [10]. Although prescribing practices are known to be influenced by factors such as the healthcare system, providers, and patients [11], Eswatini lacks specific estimates of how these factors contribute to the prevalence or incidence of medical errors. The country also continues to struggle with a high burden of infectious diseases such as HIV/AIDS and tuberculosis [12], which may further exacerbate the incidence of medical errors.

This study, therefore, aims to assess the current PSC in Eswatini’s hospitals and compare the findings with the 2018 Agency for Healthcare Research and Quality (AHRQ) survey report—a publicly available dataset comprising information from 680 hospitals [13]. The results of this study will serve as a baseline for improving PSC in Eswatini’s healthcare facilities. Additionally, the study contributes to the body of research on hospital PSC in Eswatini and has the potential to significantly enhance patient safety nationwide.

## Method

### Study setting and design

This study was conducted across five regional public government hospitals in Eswatini: Mbabane Government Hospital (MGH), Hlathikhulu Government Hospital (HGH), Lubombo Referral Government Hospital (LRGH), Piggs Peak Government Hospital (PPGH), and Mankayane Government Hospital (MkGH). Eswatini is a small country covering 17,364 km², with a population of approximately 1.2 million people [14]. The country has six government hospitals (five of which participated in this study), two mission hospitals, and around 259 primary healthcare facilities (clinics). Only a few private healthcare facilities serve a small segment of the population who can afford private care.

The sixth government hospital, Raleigh Fitkin Memorial Hospital (RFM), was excluded from the study due to its operational structure. Although built by the government, RFM is managed by a religious organization, making it distinct from the other public hospitals in terms of governance and operations.

Public hospitals are the primary providers of healthcare services in Eswatini. Therefore, surveying the PSC across the five major public hospitals is considered representative of the overall patient safety culture in the country’s healthcare system. A cross-sectional quantitative study design was used, employing the validated Hospital Survey on Patient Safety Culture (HSOPSC) developed by the Agency for Healthcare Research and Quality (AHRQ). The survey was administered to healthcare workers (HCWs) in the five participating public hospitals.

### Study population and respondents

The study population comprised healthcare workers (HCWs) from five regional hospitals, including physicians, nurses, pharmacists, support staff, technicians, and administrative personnel. These individuals were eligible to participate due to their involvement in patient care and the potential impact of their roles on healthcare safety practices within the hospitals. Frontline HCWs, in particular, are well-positioned to provide valuable insights into the prevailing safety culture [15]. HCWs from various disciplines were invited to participate, and data were collected from those who were present at the hospitals during the study period.

### Sample size and sampling

A proportional stratified sampling strategy was employed to select respondents from the five hospitals, with the number of invitations distributed based on the staff size at each facility. The researcher distributed 325 invitations across the five hospitals, which together had a total staff population of 1,487. Ultimately, 278 valid responses were received, representing an 18.69% response rate from the overall staff population.

### Data collection and measures

The study utilized the Hospital Survey on Patient Safety Culture (HSOPSC), Version 1.0, developed by the Agency for Healthcare Research and Quality (AHRQ). The questionnaire comprises 12 composites, each consisting of 3–4 items, for a total of 42 items (S1 HSOPSC Survey_ Questionnaire.pdf in S1 File). It is a validated and reliable tool for assessing patient safety culture (PSC) [15]. The questionnaire was administered in English, one of the primary languages used in Eswatini’s healthcare system.

The HSOPSC is organized into nine sections, covering various aspects such as respondent background, work area/unit, management, communication, frequency of events reported, patient safety grade, hospital characteristics, number of events reported, and additional comments. Prior to the survey launch, a team of patient safety experts reviewed the questionnaire to assess whether adjustments were needed to align it with the Eswatini context. A pilot test was then conducted at a primary healthcare facility with ten respondents to identify any necessary adaptations. The pilot results were satisfactory, and no further modifications were required for the final survey.

Cronbach’s alpha coefficients for the 12 composites ranged from 0.73 to 0.82, indicating good internal consistency and reliability. Both web-based and paper-based formats were used, depending on the healthcare worker’s preference. The surveys were conducted anonymously, with no identifiable information collected from respondents. Data collection was carried out between July 1 and September 30, 2019.

### Data analysis

Data were analyzed using SPSS (version 19) and the HSOPSC Hospital Survey Excel Tool (S2 Hospital Survey Excel Tool 1.8 VF.xlsm in S2 File) developed by AHRQ [16]. After data cleaning, descriptive statistics were conducted to report sample characteristics and assess composite scores across patient safety culture (PSC) outcome variables. The analysis involved comparing composite scores among Eswatini’s major government hospitals, evaluating positive response rates for each composite, and assessing PSC outcome ratings across the five hospitals using the AHRQ tool.

Evaluating positive response rates for each composite provided a detailed view of specific PSC dimensions, helping to identify areas that require improvement and those that are performing well. Comparing PSC outcome ratings across hospitals using the standardized AHRQ tool ensured that results could be interpreted within the broader context of international best practices and benchmarks. This analysis offers actionable insights to support future quality improvement efforts in Eswatini’s healthcare system.

Patient safety grade and frequency of events were treated as outcome variables [15], warranting more in-depth interpretation. Additionally, ANOVA was applied to determine whether there were statistically significant differences in composite PSC scores across hospitals, with significance set at p < 0.05.

To assess sample representativeness, a chi-squared test was conducted to compare the distribution of occupational roles among respondents and the overall hospital population. The AHRQ guidance was used to interpret strengths of patient safety composites—composites with higher percentages of positive responses were considered indicators of stronger PSC. To calculate a hospital’s score on a specific PSC composite, the average percentage of positive responses across all items within that composite was computed. For positively worded items, the percent positive included “Strongly agree” or “Agree” responses; for negatively worded items, “Strongly disagree” or “Disagree” responses were treated as positive.

In this study, the minimum Cronbach’s alpha score among the 12 PSC composites was 0.68, indicating an acceptable level of internal consistency. The survey also included an open-ended section for respondents to share opinions on patient safety, error reporting, or event reporting within their respective hospitals. These qualitative responses were carefully reviewed and analyzed.

### Ethical considerations

Ethical review and approval for the study were obtained from the Eswatini Health and Human Research Review Board (EHHRRB) (SHR141/2019). Permission to conduct the study was also secured from each participating hospital. Study information sheets and consent forms were distributed to inform and invite interested staff—particularly those involved in direct care services—to participate in the survey. Written informed consent was obtained from all participants prior to survey completion, through the signing of the provided consent form. Minors, vulnerable populations, and individuals lacking the capacity to provide informed consent were excluded from the study.

## Results

### Demographic characteristics of respondents

The demographic characteristics of the respondents are summarized in Table 1. The largest group consisted of nurses (n = 101, 36.33%), followed by technicians (n = 76, 27.34%). The majority of respondents were female (n = 160, 57.55%), and 71.58% were between the ages of 30 and 49. In terms of academic qualifications, most held a bachelor’s degree (n = 204, 73.38%). Additionally, more than half of the participants (n = 176, 63.31%) had been employed at their hospitals for fewer than six years. A representativeness test indicated no significant difference between the respondents’ occupations and those of the overall population (Table 2).

**Table 1:** Characteristics and demographics of respondents.

Characteristics	n	%
Gender		
Female	160	57.55%
Male	118	42.45%
Age (years)		
≥25 ≤ 29	50	17.99%
30-39	132	47.48%
40-49	67	24.10%
≥50 ≤ 65	29	10.43%
Education		
Certificate	11	3.96%
Diploma	50	17.99%
Undergraduate	204	73.38%
Master’s Degree	12	4.32%
PhD	1	0.36%
Work Area/Unit		
Medicine (non-surgical)	38	13.67%
Surgery	9	3.24%
Obstetrics	18	6.47%
Emergency department	26	9.35%
Intensive care unit (any type)	21	7.54%
Rehabilitation	12	4.32%
Diagnostics	53	19.06%
Pharmacy	17	6.12%
No specific unit	69	24.82%
Other	15	5.40%
Occupations		
Attending/ Staff physician	34	12.23%
Physician Assistant/ Nurse practitioner	28	10.07%
Registered nurse	101	36.33%
Pharmacist	15	5.40%
Technician (e.g., EKG, Lab, Radiology)	76	27.34%
Administration/ Management	7	2.52%
Other health professions	16	5.76%
Experience in hospital (years)		
<1	46	16.55%
1-5	130	46.76%
6-10	54	19.42%
11-15	15	5.40%
16-20	24	8.63%
≥21 ≤ 30	9	3.24%
Direct contact with patients		
Yes	276	85%
No	2	1%

**Table 2:** Representativeness Test.

Occupations	Respondents (samples)		Population (5 Hospitals)	
	Number	Percentage	Number	Percentage
Attending/ Staff physician	34	2.23%	159	0.69%
Physician Assistant/ Nurse practitioner	28	10.07%	155	0.42%
Registered nurse	101	36.33%	530	35.64%
Pharmacist	15	5.40%	74	4.98%
Technician (e.g., EKG, Lab, Radiology)	76	27.34%	384	25.82%
Administration/ Management	7	2.52%	57	3.83%
Other health professions	16	5.76%	128	8.61%
Total	278	100%	1487	100%

Chi−square = 4.315, p=0.6341.

### Composites and outcomes

**Fig 1:**
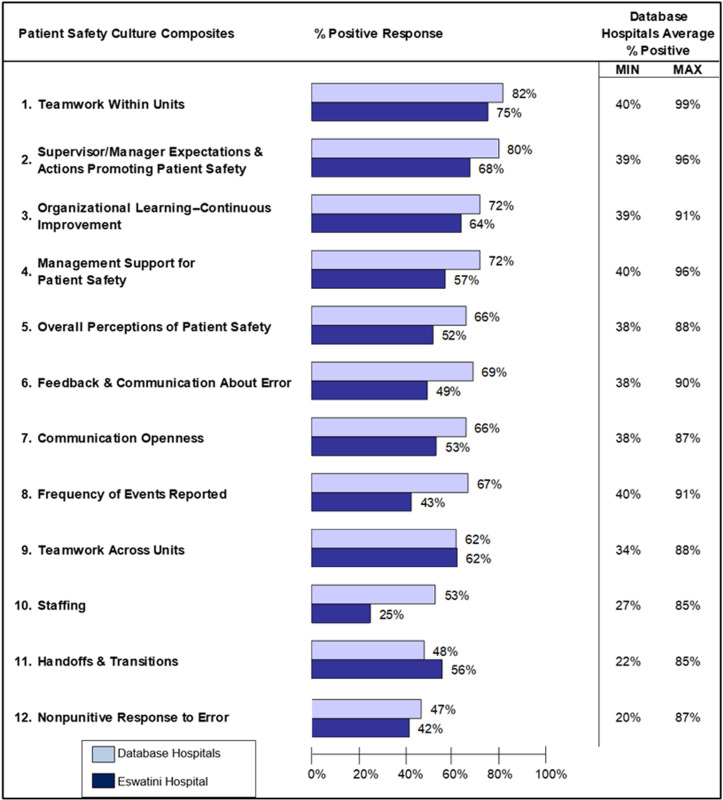
Comparative composite-level results from AHRQ (2018) in relation to Eswatini’s government hospitals [13].

The highest-rated patient safety culture (PSC) composites were teamwork within units (75%) and supervisor/manager expectations and actions promoting patient safety (68%). In contrast, the lowest-rated composites were staffing (25%), non-punitive response to error (42%), and frequency of events reported (43%). Statistically significant differences in PSC composites were observed across the five facilities (p < 0.05 for all composites except staffing), as shown in Table 3. These findings underscore the unique characteristics of PSC within each hospital. A summary of the PSC composite scores and outcome measures is presented in Table 3 and Fig 1.

**Table 3:** Survey composites – positive response rating across the five hospitals.

Composites of patient safety culture (Cronbach α)	P-value	MGH (%)	MkGH (%)	PPGH (%)	LRGH (%)	HGH (%)	Across System (%)
Number of respondents (n)		83	59	60	39	37	
Teamwork within unit (0.79)	<0.001	73	74	77	71	84	75
Supervisor/Manager Expectations & Actions Promoting Patient Safety (0.82)	<0.001	69	65	58	70	83	68
Organizational Learning— Continuous Improvement (0.73)	0.012	67	63	63	70	53	64
Hospital Management Support for Patient Safety (0.81)	<0.001	47	60	46	77	74	57
Overall Perceptions to Patient Safety (0.79)	<0.001	53	52	45	54	57	52
Feedback and Communication About Error (0.78)	<0.001	42	76	41	32	57	49
Communication Openness (0.68)	<0.001	55	49	54	46	62	53
Frequency of Events Reported (0.86)	<0.001	28	40	66	32	54	43
Teamwork Across Units (0.74)	<0.001	52	77	63	63	59	62
Staffing (0.73)	0.056	30	28	18	15	32	25
Handoffs & Transitions (0.77)	0.042	54	68	47	66	47	56
Non-punitive Response to Error (0.76)	<0.001	44	40	33	36	59	42

### Patient safety grade and number of events reported

Positive responses varied significantly among hospitals for each outcome variable, as indicated by Fisher’s exact test (p < 0.001). Despite being Eswatini’s primary referral hospital and handling the highest patient volume, Mbabane Government Hospital (MGH) reported the lowest percentage of frequently reported events (28%) and the lowest overall percentage of events reported (24.3%) compared to other hospitals. In contrast, smaller hospitals were more likely to report an “excellent” or “very good” patient safety grade than larger hospitals.

Eswatini’s overall performance was notably lower than the benchmark scores in the 2018 AHRQ report. Specifically, the “Overall Patient Safety Grade” had a percent positive response of 29%, calculated by combining respondents who rated it as “Excellent” or “Very Good.” Additionally, the “Number of Events Reported” had a 42% positive response, based on respondents who reported one or more events in the past 12 months. Both metrics were approximately 10% lower than those reported in the 2018 AHRQ benchmark, suggesting that adverse events may be underreported in Eswatini’s healthcare system. The results for the patient safety grade and number of events reported are presented in Table 4 and Fig 1.

**Table 4:** Comparison of PSC outcome ratings across the five hospitals.

Description/ Hospital	Frequency of events reported N (%)	Patient Safety Grade N (%)
**MGH**	23 (28)	17 (26)
**MkGH**	24 (40)	19 (38)
**PPGH**	40 (66)	9 (39)
**LRGH**	12 (32)	26 (26)
**HGH**	20 (54)	8 (38)
Percent positive response	119 (43)	79 (29)
P-Value	<0.001	

## Discussion

### Research findings

The findings from this study reveal that Eswatini hospitals reported a low overall positive score of 54% in patient safety culture (PSC), significantly below the average reported by the Agency for Healthcare Research and Quality (AHRQ) (Fig 1). This low PSC rating highlights the urgent need for substantial efforts to improve safety culture in Eswatini’s public hospitals. Although positive attitudes toward PSC did not vary significantly among hospitals within the country, Eswatini’s performance was similar to that of other developing African countries, such as South Africa, where studies have also reported poor-quality PSC in public hospitals [17]. However, when compared with the AHRQ’s 2018 survey, which recorded an average positive score of 65% across U.S. hospitals [13,15], Eswatini’s results were markedly lower (Table 4).

Among the PSC composites, “teamwork within units” received the highest positive score (75%), while “staffing” was rated the lowest (25%). The low staffing score underscores the urgent need for better staff retention and increased recruitment of healthcare workers (HCWs) in Eswatini’s public healthcare system. The severe staffing shortage mirrors similar findings from South African public hospitals [17] and likely contributes to the low PSC scores observed in this study. Understaffing compromises patient care and increases the risk of medical errors, as staff may resort to less effective workarounds to manage heavy workloads. These findings align with a study in Egypt, which demonstrated that increased staffing levels positively impacted PSC [18].

The “frequency of events reported” was also low in Eswatini’s hospitals, consistent with trends observed in the AHRQ report (Fig 1). Furthermore, 11 of the PSC composites in Eswatini’s hospitals fell below the benchmarks established in the AHRQ 2018 report for 630 U.S. hospitals. This pattern is comparable to findings from a study in China, which emphasized the need for targeted interventions aimed at specific sociodemographic groups to improve PSC [19]. The demographic characteristics of respondents in this study—primarily nurses, female, aged between 30 and 49, and with fewer than six years of hospital experience—may influence how PSC is perceived and addressed, and they represent a critical group for future improvement initiatives.

A significant issue identified in this study was the absence of a formal error reporting system, standardized reporting tools or templates, and a designated unit responsible for overseeing patient safety. Additionally, a prevailing culture of blame and punishment for errors inhibits the establishment of a proactive and open safety reporting environment. Respondents from two hospitals cited insufficient managerial support, with positive scores of just 47% and 46%, respectively (Table 3). One participant remarked: “There is no punctuality from the hospital’s management; they are often unavailable and fail to make sound decisions when prioritizing critical cases that may cost patients’ lives.” Despite this lack of administrative support, participants acknowledged ongoing quality improvement initiatives and customer care programs aimed at enhancing patient care. To advance PSC, it is essential for policymakers, leaders, managers, and frontline staff to prioritize patient safety as a strategic goal [20–25]. Hospital administrators should also actively engage with staff feedback to foster a safer and more efficient care environment.

Promoting a culture that encourages HCWs to report medical errors without fear of punishment is crucial. Open communication and a non-punitive approach are essential components of a high-quality healthcare system. The culture of blame remains a barrier in many countries [1], where hospitals often lack clear protocols to manage adverse events [26]. A study conducted in Chinese hospitals identified open communication, staffing, leadership support, and learning from mistakes as critical elements of a safe care culture [27]. Similarly, a punitive environment was found to significantly hinder safe hospital care [26], reflecting challenges also seen in Eswatini.

Consistent with this study, research from Southeast Ethiopia also reported low PSC scores [28]. Across many African countries—including Eswatini and South Africa—the underreporting of adverse events is a common problem. HCWs often hesitate to report incidents due to fear of punishment [29,30]. For example, in Northwest Nigeria, most nurses reported a reluctance to disclose errors, particularly when no apparent harm occurred [18]. Establishing a nationwide reporting system, along with education and a supportive work environment, is vital to increasing HCWs’ willingness to report errors and improving overall patient safety [30].

Despite the overall poor PSC ratings, there have been promising initiatives across Africa aimed at improving safety culture [25]. For instance, some organizations are developing baseline frameworks for patient safety and quality improvement that Eswatini could adopt to benchmark its own services. The Society for Quality Healthcare in Nigeria (SQHN) is one such organization focused on quality improvement and risk management in healthcare [25]. Research from countries like the Netherlands, Taiwan, and the United States has shown that hospitals with weaker PSC can benefit from learning from institutions with more mature safety cultures [31], as well as those in Poland [32]. Therefore, Eswatini’s Ministry of Health, in collaboration with hospital leadership, should make PSC a core component of quality improvement efforts and promote a non-punitive environment that encourages event reporting.

Key strategies for improvement include targeted PSC training and education for HCWs. Additionally, comparative analyses with international studies offer valuable insights into how Eswatini can enhance its PSC by learning from other healthcare systems [3,7,25,26,31–33]. These lessons can guide practical reforms and strategies to advance patient safety in Eswatini.

In conclusion, improving PSC in Eswatini’s public hospitals requires focused attention on several critical areas, including staffing, safety culture, leadership support, and the implementation of structured error reporting systems. By addressing these challenges and learning from global best practices, Eswatini can create a safer, more efficient healthcare environment for both patients and healthcare workers.

### Limitations and recommendations for further research

This study relied on self-reported surveys from healthcare workers (HCWs), which may introduce subjectivity and lack contextual validation. To enhance the robustness of future assessments, we recommend adopting objective patient safety indicators to validate the results of the PSC survey. Additionally, the data presented in this study were collected exclusively from Eswatini’s government-run public hospitals, which may limit the generalizability of the findings to non-government healthcare institutions within the country. It is also recommended that hospitals continue to administer the PSC survey regularly to track and monitor changes in safety culture over time.

To strengthen future research, investigators may consider expanding data collection and developing specific patient safety quality indicators to further validate the Hospital Survey on Patient Safety Culture (HSOPSC) findings. Furthermore, with approximately 259 primary healthcare facilities (clinics) across Eswatini, we suggest conducting the AHRQ’s Medical Office Survey on Patient Safety Culture to gain a more comprehensive understanding of safety culture within the country’s primary care sector.

## Conclusion

This study provides baseline parameters for measuring patient safety culture (PSC) in Eswatini’s public hospitals. The selected hospitals included five of the six government-run hospitals within a healthcare system largely dominated by public institutions. As such, the results are considered representative of the overall PSC situation in Eswatini’s public hospitals. The recommendations for improving PSC in Eswatini hospitals are listed below.

1 For Policymakers:

We recommend that the Eswatini Ministry of Health conduct regular, nationwide PSC surveys across all hospitals to monitor progress and identify areas requiring intervention. Additionally, as individual hospitals may lack the capacity to implement their own reporting systems, a centralized adverse event reporting system should be developed and managed at the national level to standardize incident reporting and improve the overall safety culture.

2 For Hospital Administrators:

Hospital administrators should address staffing shortages by improving staff retention and recruitment strategies, based on insights from PSC survey results. Furthermore, continuous education and training on patient safety for healthcare workers (HCWs) should be prioritized to build competence and awareness across hospital departments.

The safety culture composites from the HSOPSC emphasize the significance of overall patient safety, with low-scoring areas identified as critical targets for improvement. This study reveals that the current state of PSC in Eswatini’s public hospitals is weak, and there is an urgent need for comprehensive improvements. The five hospitals studied demonstrated ineffective and inadequate patient safety systems, with no proactive, active, or reactive mechanisms in place to address adverse events. Additionally, limited resources and insufficient managerial support were common concerns among staff.

Nevertheless, the findings from this study offer valuable insights into the status of PSC in Eswatini’s hospitals and can serve as a foundation for future research and improvement efforts. For example, a study in Egypt demonstrated that implementing the Team Strategies and Tools to Enhance Performance and Patient Safety (TeamSTEPPS) program led to significant improvements in PSC among healthcare providers [29]. Eswatini hospitals may benefit from adopting similar TeamSTEPPS training programs to foster teamwork, communication, and safety awareness among HCWs.

## Supporting information

S1 File PSC Survey_Questionnaire.pdf. (PDF)

S2 File Hospital Survey Excel Tool 1.8 VF. (XLSM)
