# Supplementary material for: Assessment of patient safety culture among healthcare employees in major Eswatini public hospitals
Source: PLoS One. 2025 Jun 25;20(6):e0325292. doi: 10.1371/journal.pone.0325292 (PMC12192286; doi:10.1371/journal.pone.0325292)
Supplement: S1 File — (PDF) [file pone.0325292.s001.pdf]

Informed Consent

Dear Participant

My name is Mpumelelo G Ndlela, a graduate student in the Taipei Medical University (Taiwan R.O.C) enrolling a Masters course in Health Care Administration who is currently doing a research study entitled,' *Assessment of Patient Safety Culture Practices in Eswatini/Swaziland Hospitals*'. You are kindly requested to participate in the research study as one of the employees of the hospital.

Purpose: This study seeks to provide a baseline for the implementation of total quality management to patient safety by assessing the current perception to the patient safety practices. How much do we care for the safety of patients in our hands?

Participation and Procedure: Participation in this research study is voluntary. The following questionnaire will require approximately 15 minutes to complete. Please answer all questions as honestly as possible and return the completed questionnaires promptly. Instructions for answering the questionnaire can be found on the form itself. For any difficulty in completing the questionnaire, please contact me directly at 76(/9)730413) or e-mail researcher at ndlelawahlubi@gmail.com to provide you with assistance. Your support and swift response in this regard will be highly appreciated.

Confidentiality and Risk: Kindly be assured that all information you provide will be strictly kept confidential and will only be used for research purposes and be informed that there is no anticipated risk by participating in this study. All data, including questionnaires will be kept in a secured place and only those directly involved with the research will have access to them. After the research is completed, the questionnaires will be destroyed.

Benefits: There are no direct benefit to participants, however this study will provide baseline information for improving working conditions for an efficient and quality services.

I have read the letter of information, heard the nature of the study explained to me and I agree to participate. All questions have been answered to my satisfaction.

Respondent' signature…………………………… Date………………………….

Hospital Survey on Patient Safety

Instructions

This survey asks for your opinions about patient safety issues, medical error, and event reporting in your hospital and will take about 10 to 15 minutes to complete.

If you do not wish to answer a question, or if a question does not apply to you, you may leave your answer blank.

- *An **"event"** is defined as any type of error, mistake, incident, accident, or deviation, regardless of whether or not it results in patient harm.*
- ***"Patient safety"** is defined as the avoidance and prevention of patient injuries or adverse events resulting from the processes of health care delivery.*

SECTION A: Your Work Area/Unit

In this survey, think of your "unit" as the work area, department, or clinical area of the hospital where you spend *most* of your work time or provide *most* of your clinical services.

What is your primary work area or unit in this hospital? Select ONE answer.

☐ a. Many different hospital units/No specific unit

☐ b. Medicine (non-surgical)

☐ c. Surgery

☐ d. Obstetrics

☐ e. Pediatrics

☐ f. Emergency department

☐ g. Intensive care unit (any type)

☐ h. Psychiatry/mental health

☐ i. Rehabilitation

☐ j. Pharmacy

☐ k. Laboratory

☐ l. Radiology

☐ m. Anesthesiology

☐ n. Other, please specify:

Age……………………………………………

Gender………………………………………..

Level of Qualification……………………….

Please indicate your agreement or disagreement with the following statements about your work area/unit.

Think about your hospital work area/unit…	Strongly Disagree ▼	Disagree ▼	Neither ▼	Agree ▼	Strongly Agree ▼
1. People support one another in this unit	☐1	☐2	☐3	☐4	☐5
2. We have enough staff to handle the workload	☐1	☐2	☐3	☐4	☐5
3. When a lot of work needs to be done quickly, we work together as a team to get the work done	☐1	☐2	☐3	☐4	☐5
4. In this unit, people treat each other with respect	☐1	☐2	☐3	☐4	☐5
5. Staff in this unit work longer hours than is best for patient care	☐1	☐2	☐3	☐4	☐5

SECTION A: Your Work Area/Unit (continued)

Think about your hospital work area/unit…

	Strongly Disagree ▼	Disagree ▼	Neither ▼	Agree ▼	Strongly Agree ▼
6. We are actively doing things to improve patient safety	☐1	☐2	☐3	☐4	☐5
7. We use more agency/temporary staff than is best for patient care	☐1	☐2	☐3	☐4	☐5
8. Staff feel like their mistakes are held against them	☐1	☐2	☐3	☐4	☐5
9. Mistakes have led to positive changes here	☐1	☐2	☐3	☐4	☐5
10. It is just by chance that more serious mistakes don't happen around here	☐1	☐2	☐3	☐4	☐5
11. When one area in this unit gets really busy, others help out	☐1	☐2	☐3	☐4	☐5
12. When an event is reported, it feels like the person is being written up, not the problem	☐1	☐2	☐3	☐4	☐5
13. After we make changes to improve patient safety, we evaluate their effectiveness	☐1	☐2	☐3	☐4	☐5
14. We work in "crisis mode" trying to do too much, too quickly	☐1	☐2	☐3	☐4	☐5
15. Patient safety is never sacrificed to get more work done	☐1	☐2	☐3	☐4	☐5
16. Staff worry that mistakes they make are kept in their personnel file	☐1	☐2	☐3	☐4	☐5
17. We have patient safety problems in this unit	☐1	☐2	☐3	☐4	☐5
18. Our procedures and systems are good at preventing errors from happening	☐1	☐2	☐3	☐4	☐5

SECTION B: Your Supervisor/Manager

Please indicate your agreement or disagreement with the following statements about your immediate supervisor/manager or person to whom you directly report.

	Strongly Disagree ▼	Disagree ▼	Neither ▼	Agree ▼	Strongly Agree ▼
1. My supervisor/manager says a good word when he/she sees a job done according to established patient safety procedures	☐1	☐2	☐3	☐4	☐5
2. My supervisor/manager seriously considers staff suggestions for improving patient safety	☐1	☐2	☐3	☐4	☐5
3. Whenever pressure builds up, my supervisor/manager wants us to work faster, even if it means taking shortcuts	☐1	☐2	☐3	☐4	☐5
4. My supervisor/manager overlooks patient safety problems that happen over and over	☐1	☐2	☐3	☐4	☐5

SECTION C: Communications

How often do the following things happen in your work area/unit?

**Think about your hospital work area/unit…**	**Never** ▼	**Rarely** ▼	**Some-times** ▼	**Most of the time** ▼	**Always** ▼
1. We are given feedback about changes put into place based on event reports	☐1	☐2	☐3	☐4	☐5
2. Staff will freely speak up if they see something that may negatively affect patient care........	☐1	☐2	☐3	☐4	☐5
3. We are informed about errors that happen in this unit........	☐1	☐2	☐3	☐4	☐5
4. Staff feel free to question the decisions or actions of those with more authority........	☐1	☐2	☐3	☐4	☐5
5. In this unit, we discuss ways to prevent errors from happening again......	☐1	☐2	☐3	☐4	☐5
6. Staff are afraid to ask questions when something does not seem right....	☐1	☐2	☐3	☐4	☐5

SECTION D: Frequency of Events Reported

In your hospital work area/unit, when the following mistakes happen, *how often are they reported?*

	**Never** ▼	**Rarely** ▼	**Some-times** ▼	**Most of the time** ▼	**Always** ▼
1. When a mistake is made, but is *caught and corrected before affecting the patient*, how often is this reported?	☐1	☐2	☐3	☐4	☐5
2. When a mistake is made, but has *no potential to harm the patient*, how often is this reported?........	☐1	☐2	☐3	☐4	☐5
3. When a mistake is made that *could harm the patient*, but does not, how often is this reported?	☐1	☐2	☐3	☐4	☐5

SECTION E: Patient Safety Grade

Please give your work area/unit in this hospital an overall grade on patient safety.

☐	☐	☐	☐	☐
**A** Excellent	**B** Very Good	**C** Acceptable	**D** Poor	**E** Failing

SECTION F: Your Hospital

Please indicate your agreement or disagreement with the following statements about your hospital.

**Think about your hospital…**	**Strongly Disagree** ▼	**Disagree** ▼	**Neither** ▼	**Agree** ▼	**Strongly Agree** ▼
1. Hospital management provides a work climate that promotes patient safety........	☐1	☐2	☐3	☐4	☐5
2. Hospital units do not coordinate well with each other........	☐1	☐2	☐3	☐4	☐5
3. Things "fall between the cracks" when transferring patients from one unit to another........	☐1	☐2	☐3	☐4	☐5
4. There is good cooperation among hospital units that need to work together	☐1	☐2	☐3	☐4	☐5

SECTION F: Your Hospital (continued)

Think about your hospital…	Strongly Disagree ▼	Disagree ▼	Neither ▼	Agree ▼	Strongly Agree ▼
5. Important patient care information is often lost during shift changes........	☐1	☐2	☐3	☐4	☐5
6. It is often unpleasant to work with staff from other hospital units	☐1	☐2	☐3	☐4	☐5
7. Problems often occur in the exchange of information across hospital units...	☐1	☐2	☐3	☐4	☐5
8. The actions of hospital management show that patient safety is a top priority...	☐1	☐2	☐3	☐4	☐5
9. Hospital management seems interested in patient safety only after an adverse event happens	☐1	☐2	☐3	☐4	☐5
10. Hospital units work well together to provide the best care for patients.....	☐1	☐2	☐3	☐4	☐5
11. Shift changes are problematic for patients in this hospital	☐1	☐2	☐3	☐4	☐5

SECTION G: Number of Events Reported

In the past 12 months, how many event reports have you filled out and submitted?

☐ a. No event reports
☐ b. 1 to 2 event reports
☐ c. 3 to 5 event reports
☐ d. 6 to 10 event reports
☐ e. 11 to 20 event reports
☐ f. 21 event reports or more

SECTION H: Background Information

This information will help in the analysis of the survey results.

1. How long have you worked in this hospital?

☐ a. Less than 1 year
☐ b. 1 to 5 years
☐ c. 6 to 10 years
☐ d. 11 to 15 years
☐ e. 16 to 20 years
☐ f. 21 years or more

2. How long have you worked in your current hospital work area/unit?

☐ a. Less than 1 year
☐ b. 1 to 5 years
☐ c. 6 to 10 years
☐ d. 11 to 15 years
☐ e. 16 to 20 years
☐ f. 21 years or more

3. Typically, how many hours per week do you work in this hospital?

☐ a. Less than 20 hours per week
☐ b. 20 to 39 hours per week
☐ c. 40 to 59 hours per week
☐ d. 60 to 79 hours per week
☐ e. 80 to 99 hours per week
☐ f. 100 hours per week or more

SECTION H: Background Information (continued)

4. What is your staff position in this hospital? Select ONE answer that best describes your staff position.

- ☐ a. Registered Nurse
- ☐ b. Physician Assistant/Nurse Practitioner
- ☐ c. LVN/LPN
- ☐ d. Patient Care Asst/Hospital Aide/Care Partner
- ☐ e. Attending/Staff Physician
- ☐ f. Resident Physician/Physician in Training
- ☐ g. Pharmacist
- ☐ h. Dietician
- ☐ i. Unit Assistant/Clerk/Secretary
- ☐ j. Respiratory Therapist
- ☐ k. Physical, Occupational, or Speech Therapist
- ☐ l. Technician (e.g., EKG, Lab, Radiology)
- ☐ m. Administration/Management
- ☐ n. Other, please specify:

5. In your staff position, do you typically have direct interaction or contact with patients?

- ☐ a. YES, I typically have direct interaction or contact with patients.
- ☐ b. NO, I typically do NOT have direct interaction or contact with patients.

6. How long have you worked in your current specialty or profession?

- ☐ a. Less than 1 year
- ☐ b. 1 to 5 years
- ☐ c. 6 to 10 years
- ☐ d. 11 to 15 years
- ☐ e. 16 to 20 years
- ☐ f. 21 years or more

SECTION I: Your Comments

Please feel free to write any comments about patient safety, error, or event reporting in your hospital.

THANK YOU FOR COMPLETING THIS SURVEY.
